# Novel fatty acyl apoE mimetic peptides have increased potency to reduce plasma cholesterol in mice and macaques[S]

**DOI:** 10.1194/jlr.M085985

**Published:** 2018-09-10

**Authors:** G. M. Anantharamaiah, David W. Garber, Dennis Goldberg, Eric Morrel, Geeta Datta, Mayakonda N. Palgunachari, Thomas C. Register, Susan E. Appt, C. Roger White

**Affiliations:** Department of Medicine,* University of Alabama at Birmingham Medical Center, Birmingham, AL 35294; LipimetiX Development, Inc.,† Natick, MA 01760; Wake Forest School of Medicine,§ Winston-Salem, NC 27157

**Keywords:** peptides, low density lipoprotein, dyslipidemia, lipoproteins, apolipoprotein E

## Abstract

Ac-hE18A-NH_2_ is a dual-domain apoE mimetic peptide that possesses the putative receptor binding domain from apoE (LRKLRKRLLR, denoted hE; residues 141–150) covalently attached to lipid-associating peptide 18A. Like apoE, Ac-hE18A-NH_2_ reduces plasma cholesterol in animal models and exhibits anti-inflammatory properties independent of its cholesterol-reducing effect. Ac-hE18A-NH_2_ has already undergone phase I clinical trials as a lipid-lowering agent. To explore the therapeutic potential more, we designed and synthesized new analogues by linking ɑ-aminohexanoic acid, octanoic acid, or myristic acid to LRRLRRRLLR-18A-NH_2_ ([R]hE18A-NH_2_) and examined the cholesterol-lowering potency in animals. The modified peptides effectively reduced plasma cholesterol in apoE-null mice fed standard chow or a Western diet; the myristyl analogue was the most effective. A single administration of the myristyl analogue reduced plasma total and LDL cholesterol in a dose-dependent manner in hypercholesterolemic cynomolgus macaques for up to 1 week despite the continuation of a cholesterol-supplemented diet. The myristyl peptide (7.4 mg/kg) reduced total and LDL cholesterol at 24 h by 64% and 74%, respectively; plasma HDL levels were modestly reduced and returned to baseline by day 7. These new analogues should exhibit enhanced potency at lower doses than Ac-hE18A-NH_2_, which may make them attractive therapeutic candidates for clinical trials.

The hepatic uptake of LDL is highly regulated. The LDL receptor (LDLR) present on hepatocytes mediates the endocytosis of LDL and thus regulates the plasma level of the lipoprotein and cholesterol (1). In the acidic environment of the endosome, the LDLR dissociates from its ligand and recycles back to the cell surface for further uptake of LDL (1). apoB is the ligand for LDLR and binds to the receptor via its receptor binding domain. apoE is an alternate ligand for the LDLR and mediates the clearance of triglyceride-rich lipoproteins such as chylomicron remnants and VLDL (2). apoE also binds to additional hepatic receptors such as LDLR-related protein and heparan sulfate proteoglycans (HSPGs) (3). Thus, even in the presence of a defective LDLR, apoE is able to clear atherogenic lipoproteins via alternate receptors.

apoE possesses two domains: the 4-helix bundle structure that contains the receptor binding domain at the N terminus and a 57-residue-long lipid-associating amphipathic helical domain (4). Based on this structure, we designed an apoE mimetic peptide by covalently linking the receptor binding domain LRKLRKRLLR (residues 141–150 from apoE) to a well-characterized 18-residue lipid-associating peptide, 18A (5). The resulting peptide, Ac-hE18A-NH_2_, associated with LDL and other atherogenic lipoproteins, altered electrophoretic mobility and enhanced LDL uptake in embryonic fibroblasts and HepG2 cells (5, 6). The uptake was not LDLR-dependent, and HSPGs were shown to be involved. These observations were further confirmed in apoE-null mice (7). The administration of the peptide to cholesterol-fed New Zealand white rabbits and Watanabe heritable hyperlipidemic rabbits also showed rapid reduction in plasma total cholesterol (TC), with the effect lasting more than 1 week after a single administration (8, 9). Furthermore, there was a concomitant increase in paraoxonase-1 and reduction in lipid hydroperoxide in the plasma of Watanabe heritable hyperlipidemic rabbits (8). Additional studies have shown that Ac-hE18A-NH_2_ ameliorated oxidative processes (9, 10), improved cognitive function, and reduced Alzheimer’s burden in a mouse model of Alzheimer’s disease (10) and reduced atherosclerotic lesion formation in apoE- and LDLR-null mice consuming Western diets (11). In humans, the peptide reduced triglyceride-rich lipoproteins, an effect that lasted for more than 1 week (9).

In our efforts to design more effective apoE mimetic peptides to reduce plasma cholesterol, we have shown that replacing lysine with arginine (Arg) in LRKLRKRLLR produced a peptide, Ac-[R]hE18A-NH_2_, that was more potent than Ac-hE18A-NH_2_ in the cellular uptake of LDL cholesterol (LDL-C) (5). This paper describes further modifications to Ac-[R]hE18A-NH_2_ that enhance plasma cholesterol clearance in hyperlipidemic mouse and monkey models. These modifications included addition of the hydrophobic amino acid Ac-ɑ-aminohexanoic acid (Ac-Aha) and fatty acid chains such as octanoic and myristic acid to the N terminus. Because the myristyl analogue of [R]hE18A-NH_2_ was the most effective in reducing plasma cholesterol, we compared this peptide to the myristyl analogue of hE18A-NH_2_. We show that the covalent addition of a myristyl group to [R]hE18A-NH_2_ or hE18A-NH_2_ to obtain Myr-LRRLRRRLLR-18A-NH_2_ and Myr-LRKLRKRLLR-18A-NH_2_, respectively, yielded peptides with almost equal potency to reduce plasma cholesterol in apoE-null mice. Myr-LRRLRRRLLR-18A-NH_2_ was also effective in reducing plasma cholesterol in monkeys.

## MATERIALS AND METHODS

### Animals

Female apoE-null mice (8 weeks old) were purchased from Jackson Laboratories (Bar Harbor, ME) and allowed a 1-week recovery period prior to initiating experimental protocols. Nonfasted mice were fed either a standard laboratory chow or a Western diet (Research Diets, Inc., New Brunswick, NJ) for 15 days prior to the study. Water was provided ad libitum. Mice were maintained at a constant humidity (60 ± 5%), temperature (24 ± 1°C), and light cycle (6 AM to 6 PM). All protocols were approved by the Institutional Animal Care and Use Committee at the University of Alabama at Birmingham and were consistent with the National Institutes of Health *Guide for the Care and Use of Laboratory Animals*. Additional studies were performed using cynomolgus macaque (*Macaca fascicularis*) monkeys (2 males and 2 females) at the Wake Forest School of Medicine. Monkeys were fed an atherogenic diet formulated to contain 32% of calories as fat and 0.29 mg cholesterol/kcal. The monkeys received 100 kcal diet/kg body weight daily prior to and during recovery from peptide administration. All procedures involving primates were performed in accordance with federal, state, and institutional guidelines and were approved by the Institutional Animal Care and Use Committee of Wake Forest University.

### Peptide synthesis

Peptides were synthesized by the solid-phase method as previously described (6). For the synthesis of Ac-Aha-LRRLRRRLLR-18A-NH_2_, fluorenylmethyloxycarbonyl-Aha was added to LRRLRRRLLR-18A-rink amide resin using the condensing agent 2-(1H-benzotriazol-1-yl)-1,1,3,3-tetramethyluronium hexafluorophosphate. After the cleavage of fluorenylmethyloxycarbonyl, the peptide was acetylated as described previously (6). The resulting peptide was designated Ac-Aha[R]hE18A-NH_2_. For the synthesis of peptides containing octanyl and myristyl end groups, five equivalents of fatty acids were dissolved in dimethylformamide and coupled to LRRLRRRLLR-18A resin using 2-(1H-benzotriazol-1-yl)-1,1,3,3-tetramethyluronium hexafluorophosphate in dimethylformamide. Myr-LRKLRKRLLR-18A resin was synthesized similarly. The peptides were cleaved from the resin along with the cleavage of *tert*-butanol-based side-chain protecting groups to yield the required acylated peptides. The peptides were purified on preparative HPLC, and purity was determined by analytical HPLC and MS analysis. The sequences of these peptides along with their chromatography retention times are summarized in **Table 1**.

**TABLE 1.** Analogues of apoE mimetic peptides

Peptide	Sequence	HPLC Peak Number^a^	Retention Time (min)
Ac-hE18A-NH_2_	Ac-LRKLRKRLLR-18A-NH_2_	1	5.67
Ac-[R]hE18A-H_2_	Ac-LRRLRRRLLR-18A-NH_2_	2	5.83
Ac-Aha-[R]hE18A-NH_2_	Ac-Aha-LRRLRRRLLR-18A-NH_2_	3	6.23
Oct-[R]hE18A-NH_2_	Octanyl-LRRLRRRLLR-18A-NH_2_	4	7.68
Myr-hE18A-NH_2_	Myristyl-LRKLRKRLLR-18A-NH_2_	5	9.07
Myr-[R]hE18A-NH_2_	Myristyl-LRRLRRRLLR-18A-NH_2_	6	9.45

Aha = NH_2_-(CH_2_)_5_-COOH; myristyl = CH_3_-(CH_2_)_12_-CO; octanyl = CH_3_-(CH_2_)_6_-CO; and 18A = DWLKAFYDKVAEKLKEAF.

a From Fig. 1.

### Isolation of human LDL

Plasma LDL was prepared from human plasma obtained from Research Blood Components (Boston, MA) by sequential ultracentrifugation (12). Plasma density was adjusted to 1.02g/ml with potassium bromide and centrifuged at 45,000 rpm in a Ti-70 ultracentrifuge rotor for 20 h. The VLDL layer was removed, and the sample was adjusted to a density of 1.063 g/ml with KBr and centrifuged at 45,000 rpm in a Ti-70 ultracentrifuge rotor for 72 h. The top LDL layer was removed and dialyzed extensively against PBS (pH 7.4) containing 1 mM EDTA. The protein content was determined by a Pierce BCA Protein Assay Kit (Thermo Fisher Scientific, Waltham, MA).

### Analytical HPLC

Analytical HPLC profiles were obtained on a C-18 reverse-phase cartridge analytical HPLC column (Interchim, Los Angeles, CA) using an acetonitrile-water gradient (35% to 70%) containing 0.1% trifluoroacetic acid for 15 min. Profiles were monitored at 220 nm.

### Agarose gel electrophoresis

Agarose gel electrophoresis was carried out according to Asztalos et al. (13) using purified human LDL fractions. LDL and the LDL peptide mixtures were electrophoresed on a 0.7% agarose gel. Tris-Tricine buffer (25 mM, pH 8.6) was used for both gel and electrode buffers. Samples of LDL (5 µL, 1 mg/ml) and LDL peptide complexes (5 µL containing 5 µg LDL and 0.5 µg of different peptides) were diluted with loading buffer (5 µl) containing 10% glycerol and bromophenol blue, electrophoresed at a constant voltage (100 V) for 30 min, and stained with Coomassie Blue. In additional experiments, plasma was collected from macaques at time points up to 7 d after the administration of Myr-[R]hE18A-NH_2_. Samples underwent agarose gel electrophoresis as previously described.

### Effect of peptides on plasma cholesterol in apoE-null mice

A blood sample was collected from apoE-null mice at baseline under isoflurane anesthesia. Mice were then randomized to receive a single dose of saline, Ac-hE18A-NH_2_, Ac-[R]hE18A-NH_2_, Ac-Aha[R]hE18A-NH_2_, Oct-[R]hE18A-NH_2_, Myr-[R]hE18A-NH_2_, or Myr-hE18A-NH_2_ (2.5–5 mg/kg) via retro-orbital injection. Oct-[R]hE18A-NH_2_ and Myr-[R]hE18A-NH_2_ were solubilized in 0.2% Tween 20 to increase the solubility of the peptides. Blood samples were collected after 1, 5, and 24 h for the measurement of plasma cholesterol. Ten microliters of cholesterol standard or plasma were added to 200 µl cholesterol reagent and incubated for 15 min at room temperature. Absorbance was monitored at 500 nm. In a separate experiment, apoE-null mice were fed a Western diet for 15 days prior to the study. Ac-hE18A-NH_2_, Ac-Aha-[R]hE18A-NH_2_, or Myr-[R]hE18A-NH_2_ was then administered at a dosage of 7.5 mg/kg to assess the effect of peptides on plasma cholesterol levels.

### Effect of peptides on plasma cholesterol in hypercholesterolemic monkeys

A vascular access port was surgically implanted in cynomolgus macaques prior to the start of the experiment. The catheters were checked regularly and maintained for patency with a saline solution containing anticoagulant and antibacterial/antifungal agents. Animals were fasted overnight for a minimum of 18 h prior to peptide administration. Monkeys were anesthetized with ketamine HCl (15 mg/kg, im) prior to dosing. Additional ketamine (∼5 mg/kg, im) was provided as needed to maintain anesthesia for 2 h after the initiation of dosing.

Monkeys received intravenous infusions of vehicle or varying doses (1, 3, 5, or 7.4 mg/kg) of Myr-[R]hE18A-NH_2_ in an escalating dose design. Each animal underwent multiple dose regimens over the course of the study and was allowed at least 3 weeks between treatments to restore cholesterol to baseline levels. Peptide solutions were formulated to allow the delivery of targeted doses on the basis of 0.35 ml/kg/min in a slow-push protocol over 10 min. During the infusion, and for at least 30 min afterward, the following parameters were monitored: heart rate, respiratory rate, PaO_2_, body temperature, and indirect blood pressure (systolic/diastolic/mean). The peptide was solubilized in normal saline (pH 7.6) containing l-Arg and Tween 20. The level of Tween 20 used corresponded to 0.2 mg Tween 20/mg active peptide. l-Arg was added at a constant ratio of 3.5 mg Arg/mg active peptide to avoid the binding of the highly positively charged peptide to sialic acid and proteoglycans on the endothelium. Prepared peptide solutions were filtered through 0.22 µm polysulfone membrane filters. Blood samples (∼1–3 ml each) were collected for each dose infusion: preinfusion and 60 min, 8 h, 24 h, 3 days, and 7 days after the initiation of infusion. TC and HDL cholesterol (HDL-C) levels were determined by the Wake Forest Comparative Medicine Clinical Chemistry and Endocrinology Laboratory using reagents (ACE cholesterol and ACE HDL-C standards) and instrumentation (ACE ALERA auto analyzer) from Alfa Wasserman Diagnostic Technologies (West Caldwell, NJ). TC and HDL-C were standardized to calibrated controls from the Centers for Disease Control and Prevention/National Institutes of Health Lipid Standardization Program. LDL-C was determined using the Friedewald equation (LDL_calc_=TC – HDL-C − TG/5) (14). TC ≥250 mg/dl was confirmed prior to the start of the study. Column lipoprotein profiles for monkey plasma were obtained according to Garber, Kulkarni, and Anantharamaiah (15).

### Statistical analysis

All results are reported as means ± SEMs. Statistical analysis was performed using SigmaStat 3.5 software. For cholesterol clearance, areas under the curve were determined by the least-squares method. Differences between groups were assessed by *t*-test or one-way ANOVA with post hoc testing (Student-Newman-Keuls test).

## RESULTS

**Fig. 1.**
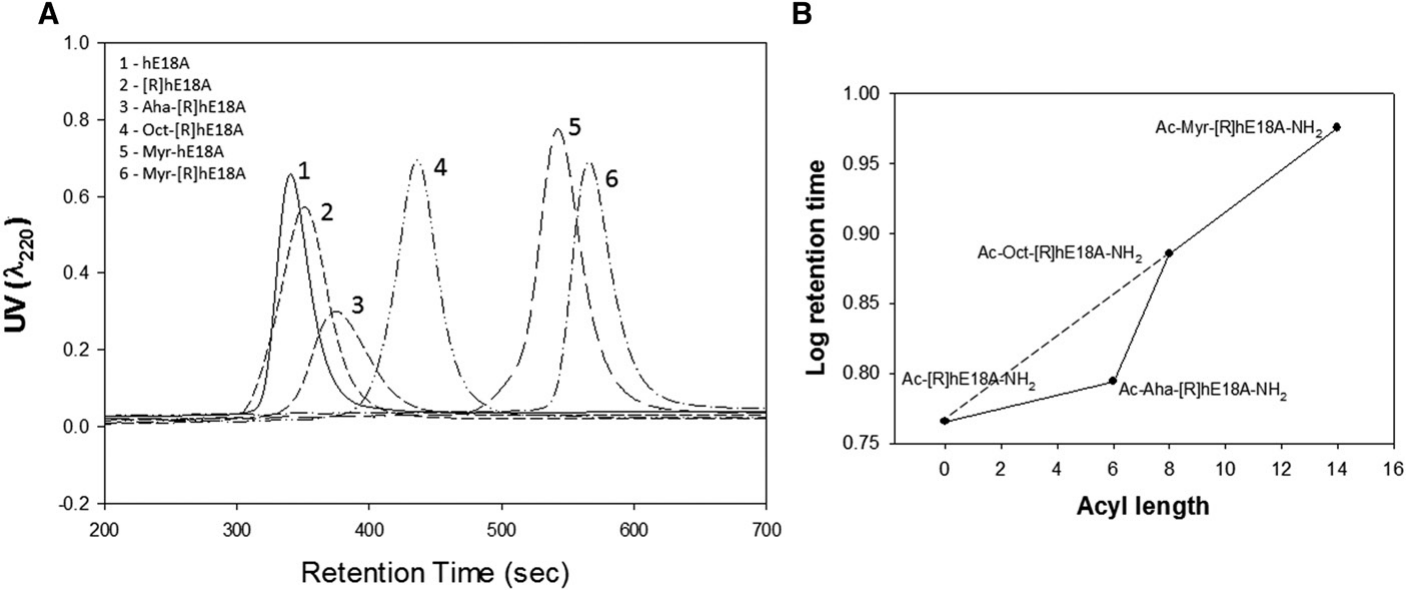
Chromatographic profiles for apoE mimetic peptides. HPLC profiles for Ac-hE18A-NH_2_, Ac-[R]hE18A-NH_2_, Ac-Aha-[R]hE18A-NH_2_, Oct-[R]hE18A-NH_2_, Myr-hE18A-NH_2_, and Myr-[R]hE18A-NH_2_. Peptides were injected on a C-18 reverse-phase column and eluted using an acetonitrile-water gradient (35% to 70%). UV absorbance was monitored at 220 nm (A). B: Plot of acyl chain length versus log retention time for Ac-[R]hE18A-NH_2_, Ac-Aha-[R]hE18A-NH_2_, Oct-[R]hE18A-NH_2_, and Myr-[R]hE18A-NH_2_.

In initial studies, we assessed analytical HPLC retention times of apoE mimetic peptides. The addition of Ac-Aha to LRRLRRRLLR-18A-NH_2_ increased its HPLC retention time compared with either Ac-hE18A-NH_2_ or Ac-[R]hE18A-NH_2_ (**Fig. 1A**). The addition of fatty acyl chains (octanyl and myristyl) to LRRLRRRLLR-18A-NH_2_ increased retention times further, indicating an increase in the hydrophobicity of these peptides (Fig. 1A). A log plot of retention time versus acyl chain length revealed a linear relationship for the peptides Ac-[R]hE18A-NH_2_, Oct-[R]hE18A-NH_2_, and Myr-[R]hE18A-NH_2_ (Fig. 1B). A lack of linearity was noted for Ac-Aha-[R]hE18A-NH_2_. The carbon backbone of this peptide is interrupted by a polar CONH group, which shifts retention time and hydrophobicity compared with the octanyl and myristyl peptides (Fig. 1B).

**Fig. 2.**
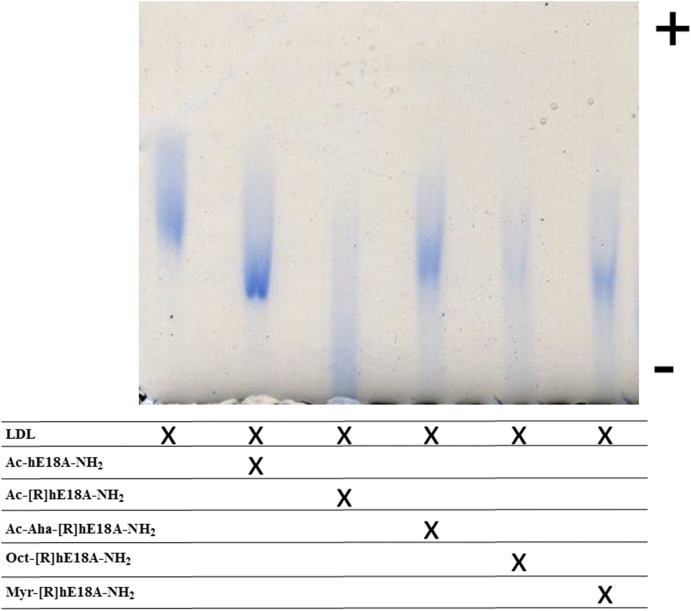
ApoE mimetic peptides alter the electrophoretic mobility of LDL. Samples of human-derived LDL (5 µg) and LDL peptide complexes were diluted with loading buffer containing 10% glycerol, loaded on an agarose gel, and subjected to electrophoresis.

In subsequent studies, apoE mimetic peptides were mixed with LDL at a 1:0.1 (w/w) ratio (based on protein) and loaded onto agarose gels. Each of the peptides retarded the mobility of LDL, thus demonstrating a physical interaction with the lipoprotein (**Fig. 2**). Compared with the LDL band, the band derived from the LDL + Ac-hE18A-NH_2_ mixture displayed a higher intensity. All of the other bands were of lower intensity, with those for Ac-[R]hE18A-NH_2_ and the octanyl derivative being diffuse. It is possible that, due to increased hydrophobicity, the incorporation of small amounts of peptides on LDL produced a prominent inhibitory effect on LDL electrophoretic mobility. This may account for the reduced band intensity for some peptide treatments.

**Fig. 3.**
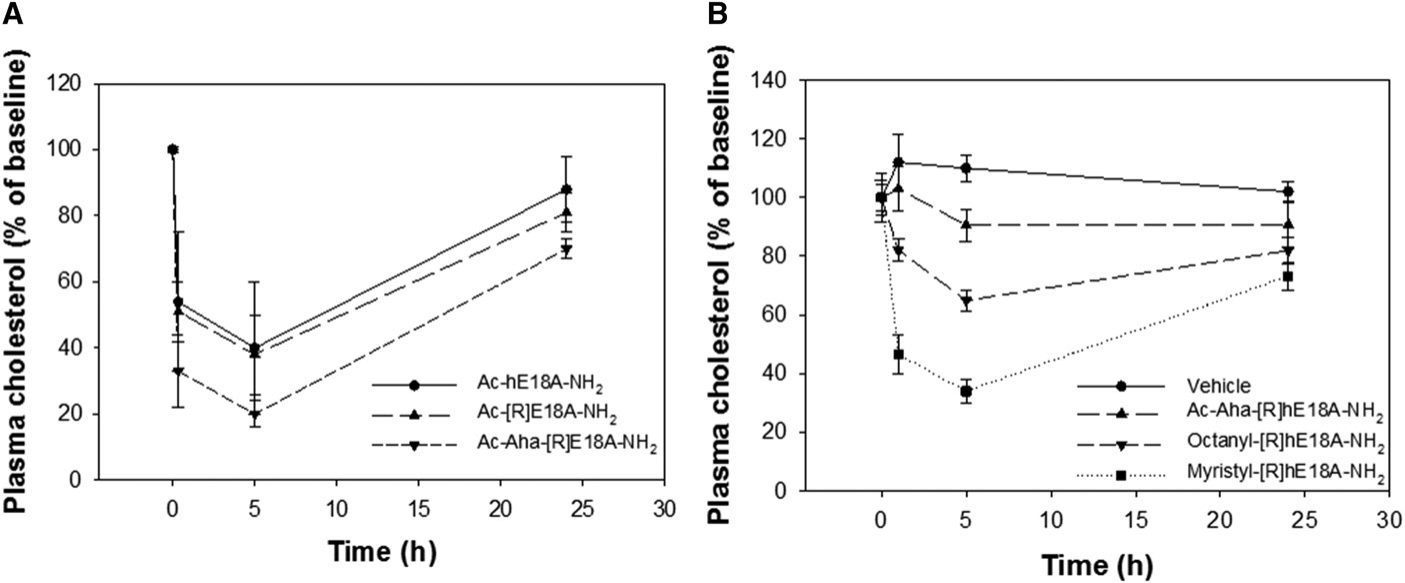
ApoE mimetic peptides reduce plasma cholesterol in apoE-null mice. A: A predose blood sample was collected from mice fed a standard chow diet, followed by retro-orbital injection of 5 mg/kg Ac-hE18A-NH_2_ (*n* = 4), Ac-[R]hE18A-NH_2_ (*n* = 4), or Ac-Aha-[R]hE18A-NH_2_ (*n* = 4). B: Cholesterol reduction in response to 2.5 mg/kg Myr-[R]hE18A-NH_2_ (*n* = 5), Oct-[R]hE18A-NH_2_ (*n* = 5), and Ac-Aha[R]hE18A-NH_2_ (*n* = 4). Data are means ± SEMs.

We previously reported that Ac-hE18A-NH_2_ was effective in reducing plasma cholesterol in different hypercholesterolemic animal models, including apoE-null mice (7–11). Plasma cholesterol levels in these mice range from 400 to 500 mg/dl when fed a standard laboratory chow. The current study utilized this model to test the efficacy of apoE mimetic peptides in reducing plasma cholesterol. Solutions of Ac-hE18A-NH_2_, Ac-[R]hE18A-NH_2_, and Ac-Aha[R]hE18A-NH_2_ were prepared in sterile saline and administered to mice at a dose of 5 mg/kg. Under these conditions, Ac-Aha[R]hE18A-NH_2_ was more effective in reducing cholesterol levels than the extensively studied Ac-hE18A-NH_2_ or Ac-[R]hE18A-NH_2_ (**Fig. 3A**). HPLC studies show that the covalent addition of Ac-Aha, octanoic acid, and myristic acid to the peptide sequence LRRLRRRLLR-18A-NH_2_ increased its hydrophobicity with the following rank order: Myr-[R]hE18A-NH_2_ > Oct-[R]hE18A-NH_2_ > Ac-Aha[R]hE18A-NH_2_ (Table 1). On this basis, we hypothesized that acylated peptides may more readily associate with atherogenic lipoproteins to lower plasma cholesterol. Thus, anticipating a higher potency of Oct-[R]hE18A-NH_2_ and Myr-[R]hE18A-NH_2_, peptides were administered at a dose of 2.5 mg/kg to apoE-null mice fed normal chow. Results show that the acyl peptides with an increasing chain length showed enhanced ability to reduce plasma cholesterol compared with Ac-Aha[R]hE18A-NH_2_ (Fig. 3B)_._

**Fig. 4.**
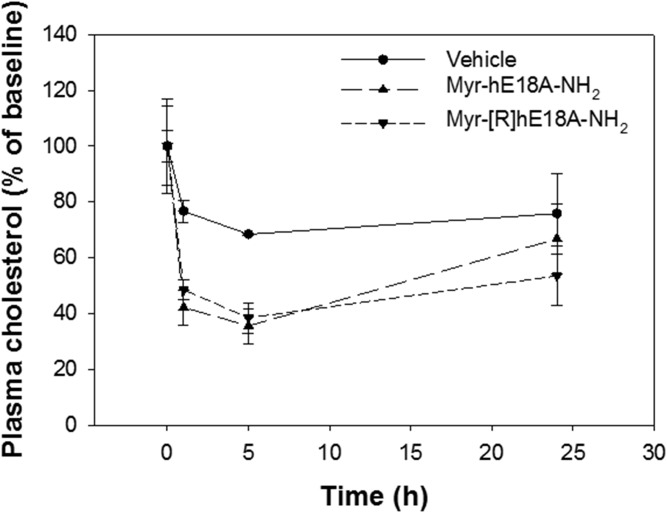
Effects of myristylated peptides on plasma cholesterol in apoE-null mice. Myr-hE18A-NH_2_ (*n* = 3) or Myr-[R]hE-18A-NH_2_ (*n* = 3) was injected in mice at a dose of 2.5 mg/kg. Both myristylated analogues induced a similar reduction in plasma cholesterol. Control mice (*n* = 3) received 0.2% Tween 20 as vehicle treatment. Data are means ± SEMs.

The administration of Oct-[R]hE18A-NH_2_ reduced plasma cholesterol by approximately 35% at 5 h. The myristylated peptide was the most effective, however, with >60% reduction in plasma cholesterol 5 h after peptide administration. Both acyl peptides were more effective in reducing plasma cholesterol than Ac-Aha[R]hE18A-NH_2_ (Fig. 3B). We also compared the abilities of a myristyl derivative of LRKLRKRLLR-18A-NH_2_ (Myr-hE18A-NH_2_) and Myr-[R]hE18A-NH_2_ to reduce plasma cholesterol. Both the peptides dramatically reduced plasma cholesterol in a dose-dependent manner. The addition of the myristyl group to both LRKLRKRLLR-18A-NH_2_ and LRRLRRRLLR-18A-NH_2_ enhanced plasma cholesterol clearance compared with saline control. Both peptides displayed a similar dose-dependent reduction in plasma cholesterol 1 and 5 h after administration (**Fig. 4**).

**Fig. 5.**
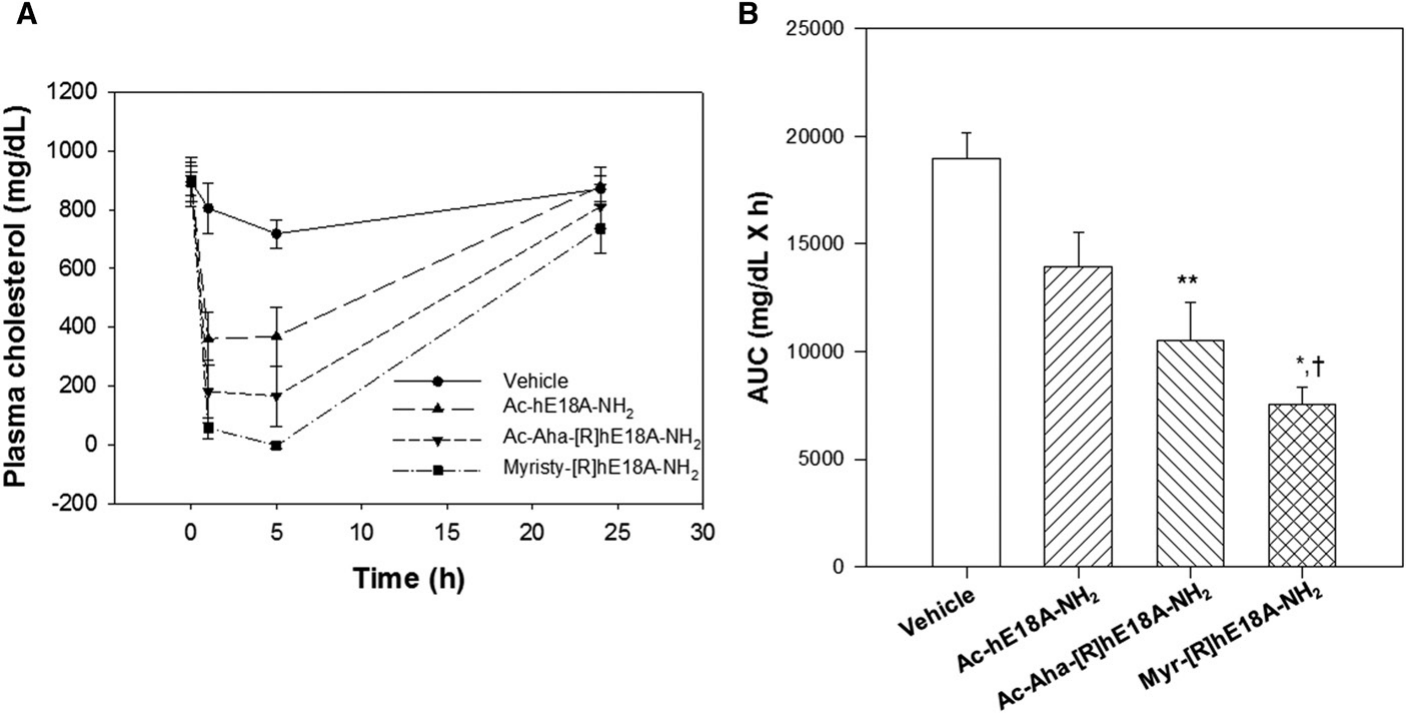
Efficacy of apoE mimetic peptides in dyslipidemic apoE-null mice. A: ApoE mimetic peptides (7.5 mg/kg) were injected in apoE-null mice fed a Western diet. Cholesterol reduction was monitored in response to vehicle control (*n* = 4), Ac-hE18A-NH_2_ (*n* = 5), Ac-Aha-[R]hE18A-NH_2_ (*n* = 5), and Myr-[R]hE18A-NH_2_ (*n* = 5). B: Changes in plasma cholesterol over the 24 h study period were quantitated by measuring the AUCs by the least-squares method for each animal from panel A. Data are means ± SEMs. *P < 0.02 versus Ac-hE18A-NH_2_; **P < 0.01 versus control; and ^†^*P* < 0.001 versus control. AUC, area under the curve.

Because the myristyl derivative of [R]hE18A-NH_2_ was highly effective in reducing plasma cholesterol in apoE-null mice fed a normal diet, we assessed the cholesterol-lowering response to Ac-hE18A-NH_2_, Ac-Aha-[R]hE18A-NH_2_, and Myr-[R]hE18ANH_2_ in apoE-null mice fed a Western diet. (**Fig. 5**). Western diet consumption increased plasma cholesterol levels in apoE-null mice to approximately 900 mg/dl. Despite the development of severe dyslipidemia in these mice, the administration of Myr-[R]hE18A-NH_2_ cleared most of the plasma cholesterol at the 5 h time point. We measured the area under the curves as an index of overall cholesterol reduction (Fig. 5B). Myr-[R]hE18A-NH_2_ was almost twice as effective as Ac-hE18A-NH_2_ in reducing plasma cholesterol over the 24 h study period. Similar to results in apoE-null mice fed normal chow, Ac-Aha-[R]hE18A-NH_2_ showed a trend of being more effective in reducing plasma cholesterol than the extensively studied Ac-hE18A-NH_2_ (Fig. 5A).

**Fig. 6.**
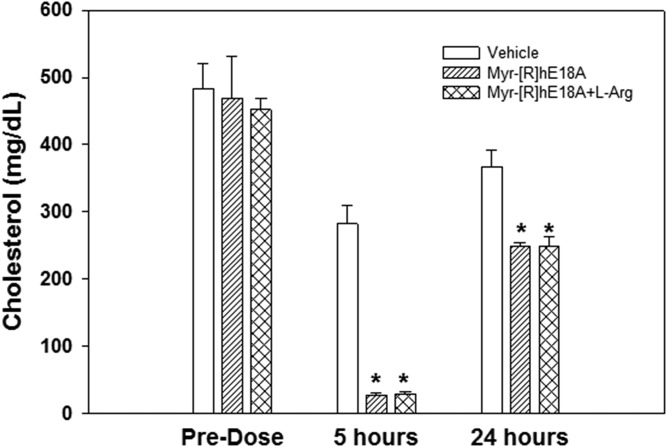
l-Arg decoy does not influence peptide-mediated cholesterol reduction in apoE-null mice. Myr-[R]hE18A-NH_2_ was solubilized in Tween 20 in the presence of l-Arg to prevent the binding of the peptide at the injection site. The peptide was also prepared in the absence of l-Arg. Each peptide solution was injected at a dose of 2.5 mg/kg in apoE-null mice, and the effects on plasma cholesterol were monitored. Data are means ± SEMs (*n* = 5 per treatment). *P < 0.001 versus saline control.

Effects of apoE mimetic peptide administration on plasma cholesterol were also tested in hypercholesterolemic cynomolgus macaques. Hypercholesterolemia was induced in monkeys by feeding a cholesterol-supplemented Western diet for at least 12 weeks prior to dosing. Mean TC and LDL-C levels were approximately 300 and 225 mg/dl in these animals, respectively. Because Myr-[R]hE18A-NH_2_ showed the greatest cholesterol-lowering effect in apoE-null mice, this mimetic was selected for evaluation in monkeys. In monkey studies, to avoid the binding of highly positively charged peptide to sialic acid and proteoglycans at the site of administration, we used l-Arg as a decoy at doses of peptide >2.5 mg/kg. To confirm that the addition of Arg did not interfere with the plasma cholesterol reduction effect of the peptide, peptide solutions in Tween 20 and Tween 20 plus l-Arg were administered in apoE-null mice. The addition of l-Arg resulted in a similar reduction of plasma cholesterol (**Fig. 6**), thus confirming that l-Arg has no effect on the ability of the peptide to reduce plasma cholesterol.

**Fig. 7.**
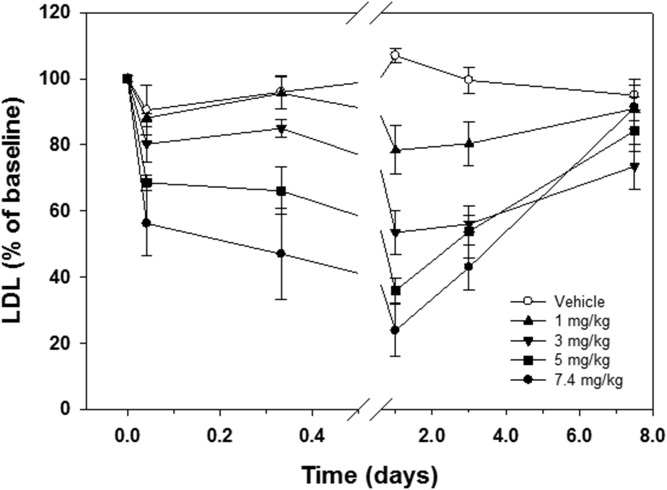
Myr-[R]hE18A-NH_2_ reduces LDL in cynomolgus monkeys. Plasma LDL concentration was measured at baseline and at time periods up to 7 d after the intravenous administration of increasing doses of Myr-[R]hE18A-NH_2_. Changes in LDL at each time postdose were measured relative to predose LDL concentrations. Data are means ± SEMs (*n* = 4 per treatment). Microsoft Excel was used to first perform a single-factor ANOVA comparing the mean 24 h LDL minimums following administration at each dose level. That analysis rejected the hypothesis that all of those minimums were equal to one another. A follow-up Newman-Keuls multiple-range test was then performed to test all possible pairwise comparisons for any significant differences between minimum mean LDL levels following each dose. Based on four monkeys per group to calculate the mean minimum LDL levels, the Newman-Keuls analysis determined that all minimums were significantly different from one another at the *P* < 0.025 level.

**Fig. 8.**
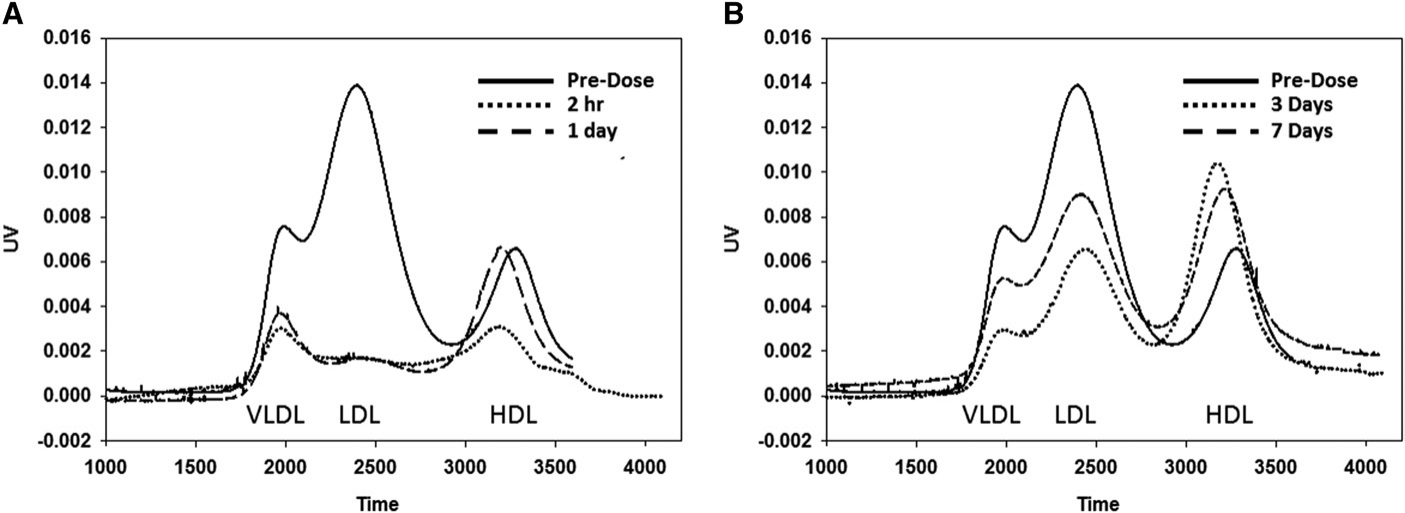
Effect of Myr-[R]hE18A-NH_2_ on the cholesterol profiles at different times postadministration in a cynomolgus monkey. Lipoprotein profiles for a monkey receiving Myr-[R]hE18A-NH_2_ at a dose of 7.4 mg/kg at time points up to 1 day (A) and 7 days (B) after peptide administration. Plasma samples (100 uL) were injected onto a Superose column. PBS (0.4 ml/min) was used to elute the samples. Cholesterol reagent (0.2 ml/min) was then mixed with the eluent, followed by heating to 57°C and measurement of UV absorbance at 540 nm.

Myr-[R]hE18A-NH_2_ was administered to hypercholesterolemic monkeys at different doses (1.0–7.4 mg/kg), and blood was collected at intervals over a 1 week time period. At the highest administered dose (7.4 mg/kg), the peptide significantly reduced TC within 8 h after administration (**Table 2**). This effect was maintained through day 3 and returned to predose levels by day 7. A reduction in LDL-C followed a similar time course (**Fig. 7**, Table 2). By day 7, LDL-C was similar to that observed in monkeys treated with vehicle (Fig. 7). The electrophoretic mobility of LDL isolated from monkeys receiving 7.4 mg/kg Myr-[R]hE18A-NH_2_ was tested. LDL mobility was reduced in samples drawn within 30 min after peptide administration, and this response was maintained after 24 h (supplemental Fig. S1). The apoB content of plasma samples was also monitored by agarose and SDS-PAGE. A reduction in apoB levels mirrored changes in LDL concentration over this time period (supplemental Fig. S2A, B). There was a trend for a reduction in HDL-C levels and plasma apoA-I content over the study period, but this did not reach significance (Table 2, supplemental Fig. S2C). The plasma cholesterol column profiles from one of the monkeys showed that even after 7 days, atherogenic LDL-C was significantly reduced after one administration of peptide (**Fig. 8A**, B). In this animal, there was a rapid rebound in HDL levels, with HDL-C equal to or exceeding baseline levels 3 days after peptide administration (Fig. 8B).

**TABLE 2.** Time-dependent changes in plasma lipoproteins in cynomolgus macaques treated with 7.4 mg/kg Myr-[R]hE18A-NH_2_.

Time	TC	LDL	HDL-C	Triglycerides
	*mg/dl*			
Predose	296 ± 38	223 ± 47	60 ± 14	68 ± 14
1 h	190 ± 42	135 ± 43	42 ± 8	67 ± 16
8 h	154 ± 40*	105 ± 34	35 ± 10	70 ± 14
1 day	108 ± 22*	58 ± 22*	33 ± 9	83 ± 19
3 days	142 ± 14*	87 ± 10*	41 ± 6	72 ± 21
7 days	271 ± 40	205 ± 43	54 ± 8	69 ± 5

* *P* < 0.05 versus predose level.

## DISCUSSION

Despite the extensive use of statins to lower plasma LDL, this approach is not sufficient to markedly decrease plasma cholesterol in all subjects. Statins are not sufficiently effective in homozygous and heterozygous familial hypercholesterolemic patients. Furthermore, several undesirable side effects of this class of drug have been reported. Recently, proprotein convertase subtilisin type 9 (PCSK-9) has been developed as a therapy to reduce plasma cholesterol. By inhibiting PCSK-9-mediated degradation of cell-surface LDL receptors, plasma cholesterol can be effectively decreased (16, 17). Anti-PCSK-9 therapy is dependent on a functional LDLR as well as the ligand. In contrast, because apoE can bind to several receptors, including HSPGs, large amounts of all of the atherogenic lipoproteins can be cleared by bypassing the LDLRs (18). Our data show that apoE mimetic peptides interact with different lipoprotein fractions in mice and monkeys. The major cholesterol fraction in apoE-null mice is VLDL-like in size but is enriched in cholesterol rather than triglycerides. In contrast, LDL is the principal cholesterol-containing particle in cynomolgus macaques. Thus, there is a fundamental difference in lipoprotein metabolism between these two species. The phospholipid layer present in different lipoprotein particles represents a common target, however, for the binding of apoE mimetics with atherogenic lipoproteins. This interaction appears to be critical for the clearance of circulating lipoproteins.

Furthermore, native apoE exerts direct anti-inflammatory/anti-atherogenic effects on several cell types in the vessel wall, immune system, and bone marrow (19). These effects are independent of its ability to clear atherogenic lipoproteins from circulation. ApoE mimetic peptides have also been shown to dramatically reduce plasma cholesterol via HSPG receptors (6–9). They also exhibit anti-inflammatory effects that are independent of their ability to reduce plasma cholesterol (18). Analogous to apoE, the peptide Ac-hE18A-NH_2_ also undergoes cellular recycling (20). Although the peptide is highly effective, we sought to improve its design with the aim of enhancing the ability to reduce plasma cholesterol with lower doses of peptide. Table 1 shows the sequences of the newly designed peptides.

Because earlier results showed replacing Lys in LRKLRKRLLR with Arg resulted in Ac-[R]hE18A-NH_2_ with enhanced potency to clear LDL in HepG2 cells (7), we used this peptide as a template to design newer peptides with higher potency. All of the peptides associated with LDL and modified LDL electrophoretic mobility (Fig. 2). Because the peptides are positively charged, these cationic peptides retard the electrophoretic mobility of LDL. Ac-Aha is an unnatural amino acid, and the addition of this group at the N terminus of the peptide increases the hydrophobicity of the resulting peptide significantly, as supported by the HPLC profile (Fig. 1). This modification resulted in the enhanced ability of this peptide to reduce plasma cholesterol in apoE-null mice compared with either Ac-hE18A-NH_2_ or Ac-[R]hE18A-NH_2_ (Fig. 3A). Encouraged by this, we sought to increase the hydrophobicity of Ac-[R]hE18A-NH_2_ by replacing Ac at the N terminus of LRRLRRRLLR-18A-NH_2_ with octanyl and myristyl groups (Table 1). As expected, the resulting peptides showed increased reverse-phase HPLC retention times with an increase in the fatty acyl chain length (Fig. 1). The deviation from this linearity in retention time of Ac-Aha-[R]hE18A-NH_2_ (Fig. 1B) can be explained by the fact that the N terminus of this peptide is CH_3_-CONH-(CH_2_)_5_-CO-NH instead of CH_3_-(CH_2_)_6_-CO-NH and CH_3_-(CH_2_)_12_-CO-NH for octanyl and myristyl peptide, respectively.

At a dose of 5 mg/kg, Ac-Aha-[R]hE18A-NH_2_ reduced plasma cholesterol by 80% at a time point 5 h after administration. To properly compare the efficacies of different acyl peptides, we studied the ability of these modified peptides to reduce plasma cholesterol in apoE-null mice at a 2.5 mg/kg dose. As can be seen in Fig. 3B, both the peptides were more effective than Ac-Aha-[R]hE18A-NH_2_. Myr-[R]hE18A-NH_2_ was the most effective and probably two times more effective than the original Ac-hE18A-NH_2_, decreasing plasma cholesterol to an almost undetectable amount at 5 h. Fig. 4 shows that the myristyl derivative of hE18A-NH_2_ (Myr-hE18A-NH_2_) was almost as effective as Myr-[R]hE18A-NH_2_.

Given the efficacy of the peptide Myr-[R]hE18A-NH_2_, we wanted to compare the cholesterol-lowering ability of this peptide to the original Ac-hE18A-NH_2_ and Ac-Aha-[R]hE18A-NH_2_ under severe dyslipidemic conditions. Thus, we selected a female apoE-null mouse model using the Western diet. The plasma cholesterol of apoE-null mice that were fed a Western diet for 15 days increased from approximately 450 mg/dl to close to 1,000 mg/dl. Even under these extreme dyslipidemic conditions, the administration of Myr-[R]hE18A-NH_2_ reduced plasma cholesterol to almost negligible levels at 5 h (Fig. 5A). As shown in Fig. 5B, Myr-[R]hE18A-NH_2_ was almost twice as effective as Ac-hE18A-NH_2_ in reducing plasma cholesterol over the 24 h study period. Similarly, Myr-[R]hE18A-NH_2_ at a dose of 7.4 mg/kg reduced plasma LDL in cynomolgus monkeys by approximately 75% after 24 h, whereas 12 mg/kg Ac-hE18A-NH_2_ was required to elicit the same response (supplemental Fig. S3). Therefore, these results indicate that the peptide Myr-[R]hE18A-NH_2_ is the most effective analogue yet described for reducing plasma cholesterol under severe dyslipidemic conditions.

To further explore the cholesterol-lowering capacity of the myristyl analogue (and with the aim of developing a strategy to study this in humans), we compared the efficacy of this peptide with Ac-hE18A-NH_2_, which has already undergone tolerability and toxicity tests in patients with dyslipidemia (9). In these initial clinical studies, Ac-hE18A-NH_2_ administration was shown to suppress plasma triglyceride levels below baseline for more than a week. Cynomolgus monkeys were fed a Western diet to increase the circulating plasma cholesterol levels. Monkeys received an infusion of Myr-[R]hE18A-NH_2_ containing 1, 3, 5, or 7.4 mg/kg peptide. The peptide decreased plasma cholesterol in a dose-dependent manner (Fig. 7C) despite continuous administration of a cholesterol-enriched diet. At all doses, TC and LDL-C levels were below baseline even 3 days after peptide administration (Fig. 7C). Figure 8A and show the plasma lipoprotein profile of a representative monkey at earlier and later time points at a 7.4 mg/kg dose of Myr-[R]hE18A-NH_2_. Column lipoprotein profiles showed LDL-C being lowest 24 h after peptide administration (38 mg/dl compared with 197 mg/dl at baseline). Even 1 week after peptide administration, LDL-C was significantly lower (114 mg/dl) compared with baseline. Lipoprotein profiles show that HDL-C was reduced up to 1 day after peptide administration (Fig. 8A). By day 3, HDL-C levels rebounded (Fig. 8B). The administration of Myr-[R]hE18A-NH_2_ retarded the electrophoretic mobility of LDL on agarose gels. This response returned to baseline by 24 h after peptide administration (supplemental Fig. S1). This suggests a physical interaction between the peptide and circulating lipoproteins and reinforces our observation that in vitro mixing of apoE mimetics with purified LDL alters characteristics of the lipoprotein. The mechanism for this prolonged effect of the peptide is unclear but may be due to either altered lipoprotein synthesis or the facilitated release of cell-surface-bound apoE from macrophages. Thus, apoE mimetic peptide administration may induce a prolonged decrease in circulating lipoproteins via multiple mechanisms.

Our results indicate that the new apoE analogue Myr-[R]hE18A-NH_2_ is the most effective agent yet described for dramatically lowering plasma cholesterol even under severe dyslipidemic conditions. Cholesterol-lowering drugs that work through the upregulation of the LDLR, such as statins, bile acid sequestrants, and PCSK-9 inhibitors, are ineffective for lowering plasma cholesterol in familial hypercholesterolemic patients. In these patients, plasma apheresis is considered the last treatment option, particularly when treatment targets are not met (21). Recently, lipoprotein apheresis has been shown to affect lipoprotein-particle subclasses more efficiently compared with the PCSK-9 inhibitor evolocumab (22). Considering apoE mimetics have been shown to enhance paraoxonase-1 activity, reduce plasma lipid hydroperoxides, exert potent anti-oxidant properties, and dramatically reduce plasma cholesterol, the new potent apoE mimetics described in this study may provide an alternative to plasma apheresis, a procedure not well tolerated in all patients. In addition, given that Ac-hE18A-NH_2_ was effective in several lipid-mediated inflammatory diseases such as Alzheimer’s disease (8, 10), the new potent analogues may represent effective alternative treatment strategies in several lipid-mediated inflammatory diseases. In support of this, we recently reported that Ac-Aha-[R]hE18A-NH_2_ exerts prominent anti-inflammatory and anti-apoptotic effects in lipopolysaccharide-treated THP-1 macrophages (23). Ongoing studies are testing the anti-inflammatory properties of Oct-[R]hE18A-NH_2_ and Myr-[R]hE18A-NH_2_ under in vitro and in vivo conditions.

## Supplementary Material

Supplemental Data
