# Supplementary material for: Novel fatty acyl apoE mimetic peptides have increased potency to reduce plasma cholesterol in mice and macaques
Source: J Lipid Res. 2018 Sep 10;59(11):2075–83. doi: 10.1194/jlr.M085985 (PMC6210918; doi:10.1194/jlr.M085985)
Supplement: Supplemental Data [file supp_59_11_2075__index.html]

Novel Fatty Acyl ApoE Mimetic Peptides Have Increased Potency To Reduce Plasma Cholesterol In Mice And Macaques — Novel Fatty Acyl ApoE Mimetic Peptides Have Increased Potency To Reduce Plasma Cholesterol In Mice And Macaques — Novel fatty acyl apoE mimetic peptides have increased potency to reduce plasma cholesterol in mice and macaques — Supplemental Data 

# Novel fatty acyl apoE mimetic peptides have increased potency to reduce plasma cholesterol in mice and macaques

## Supplemental Data

- Supplemental Figures (.pdf, 221 KB) - Supplemental Figures
