# Supplementary material for: Novel fatty acyl apoE mimetic peptides have increased potency to reduce plasma cholesterol in mice and macaques
Source: J Lipid Res. 2018 Sep 10;59(11):2075–83. doi: 10.1194/jlr.M085985 (PMC6210918; doi:10.1194/jlr.M085985)
Supplement: Supplemental Data [file 10.1194_M085985_jlr.M085985-1.pdf]

Supplemental Data

Novel Fatty Acyl ApoE Mimetic Peptides Have Increased Potency To Reduce Plasma Cholesterol In Mice And Macaques

Gattadahalli M. Anantharamaiah, David W. Garber, Dennis Goldberg, Eric Morrel, Geeta Datta, Mayakonda Palgunachari, Thomas C. Register, Susan E. Appt, and C. Roger White

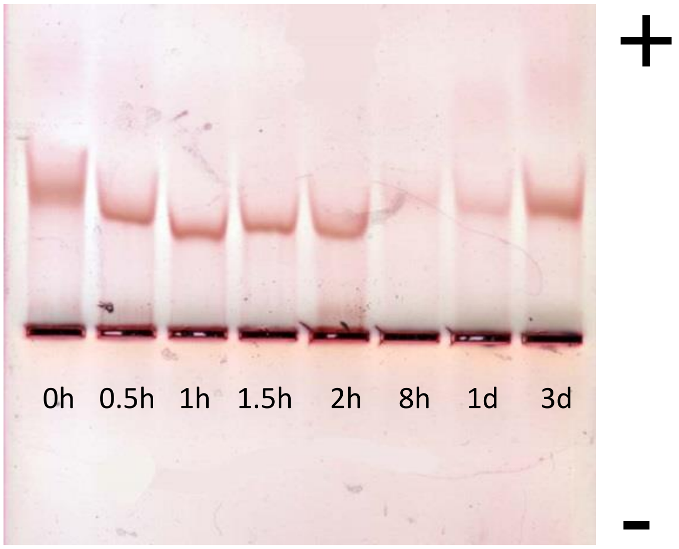

Supplemental Figure S1: Myr-[R]hE18A-NH_2 retards the electrophoretic mobility of LDL isolated from cynomolgus monkeys. Monkeys received an IV infusion of Myr-[R]hE18A-NH_2 (7.4mg/kg). Blood was collected at baseline and at time points up to 3d after peptide administration. Plasma samples were diluted with loading buffer containing 10% glycerol, loaded on an agarose gel, subjected to electrophoresis and stained with oil red O.

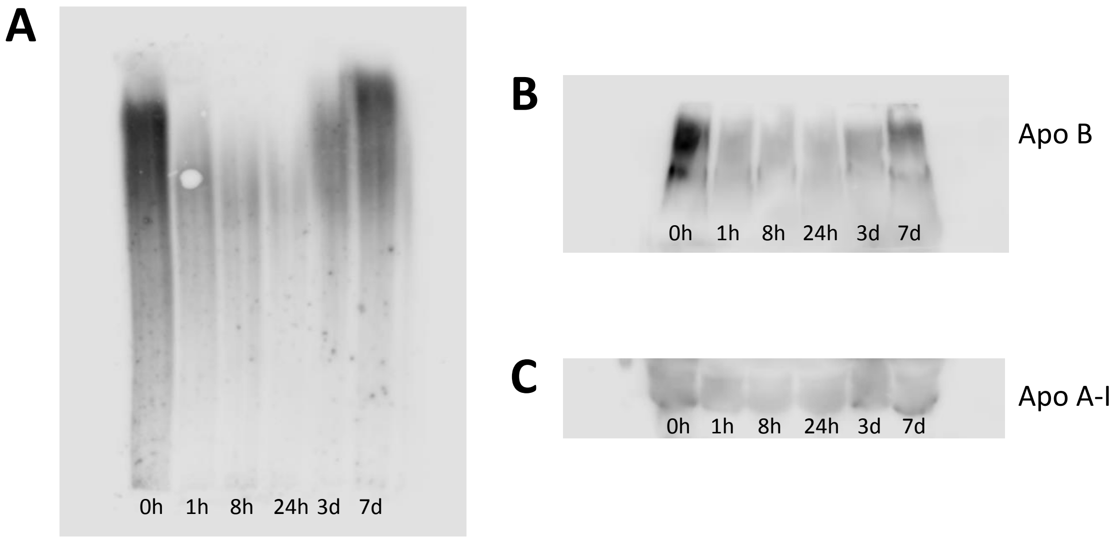

Supplemental Figure S2: Effects of Myr-[R]hE18A-NH_2 on apolipoprotein levels in plasma of cynomolgus monkeys. Plasma samples obtained at baseline and after injection of the peptide were loaded on an agarose gel. Proteins were transferred to nitrocellulose membranes and probed for apoB **(Panel A)**. Plasma samples were also resolved on SDS gels. ApoB protein was reduced after peptide administration and returned to baseline after 7d (Panel B). A modest, but non-significant, reduction in apoA-I levels mirrored changes in plasma HDL levels over this time period (Panel C).

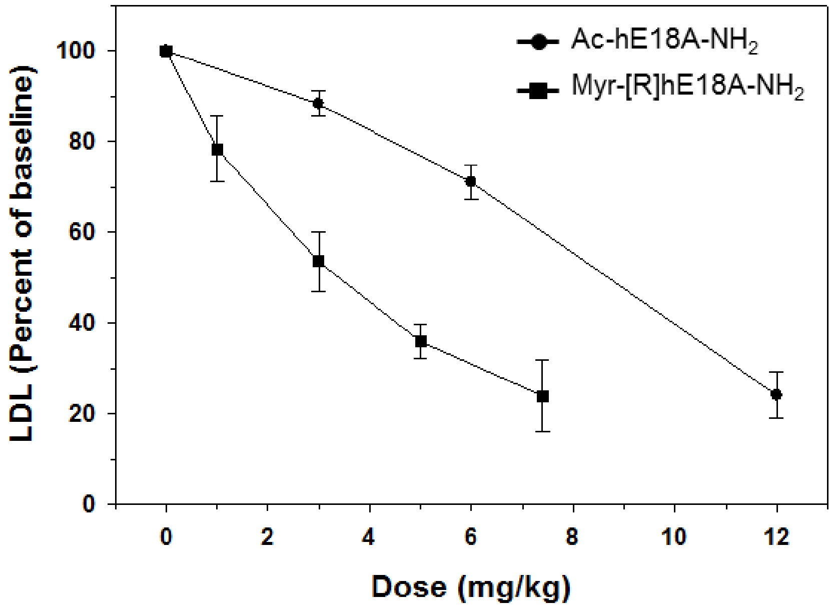

Supplemental Figure S3: Myr-[R]hE18A-NH_2 is more potent than Ac-hE18A-NH_2 in reducing plasma LDL. Plasma samples were collected from cynomolgus monkeys 24h after administration of Myr-[R]hE18A-NH_2 (0-7.4mg/kg) or Ac-hE18A-NH_2 (0-12mg/kg). 7.4mg/kg Myr-[R]hE18A-NH_2 elicited an approximate 75% reduction in LDL while 12mg/kg Ac-hE18A-NH_2 was required for this response. N=4 per peptide dose.
